# A Dual Perspective on Retracted Articles: Implications for Scientific Integrity

**DOI:** 10.34172/jrhs.13863

**Published:** 2026-08-15

**Authors:** Ali Reza Soltanian

**Affiliations:** ^1^Modeling of Noncommunicable Diseases Research Center, Hamadan University of Medical Sciences, Hamadan, Iran

 The retraction of published scientific articles has emerged as a significant and escalating challenge within our nation’s research ecosystem,^1^ compelling a critical question for academic bodies: What stance should scientific institutions adopt concerning these retracted publications and their associated authors?

 To address this issue, it is required to establish a precise definition of a retracted article. Retraction signifies that a previously published paper has been officially declared scientifically invalid, either through the initiative of the author(s) or by the journal’s editorial board. More precisely, the article is essentially rendered *null and void* by the publishing entity. Interested readers can refer to the guidelines of the Committee on Publication Ethics (COPE) for more information.^2^

 There are multiple validated reasons for article retraction, one of which is self-retraction or voluntary withdrawal by the researcher(s).^3^ It is crucial to recognize that authors retracting their work post-publication profoundly contribute to scientific transparency. In addition, a robust mechanism must be established in instances where further investigation or broader scholarly discourse reveals errors or deviations in the published findings. This mechanism should be championed by legislative and scientific bodies to incentivize authors to retract their work courageously, rather than deterring them due to the fear of legal sanctions or irreversible damage to their professional standing.

 To the best of our knowledge, no comprehensive data are currently available regarding the prevalence of published findings that are scientifically unsound and warrant retraction. Many researchers may refrain from initiating withdrawal due to apprehension over career repercussions within both national and international academic spheres. Moreover, it must be stressed that such compromised articles risk continuous citation by other researchers, potentially steering subsequent research endeavors down erroneous pathways for many years.

## Contextualizing Retraction: Evolution Versus Misconduct

 It is imperative to note that a subset of retracted studies, although conducted with sound initial methodology, may lose scientific validity over time. This can occur due to longitudinal patient follow-up, expanded cohort studies, broader geographical sampling, increasing sample size, or the advent of superior equipment and evolving technology. In these specific circumstances, the invalidation of prior findings, whether initiated by the authors or the journal, is an inherent characteristic of empirical investigation. Nonetheless, retraction in these cases should ‘not’ result in the marginalization of the author(s) from scientific discourse.

 Conversely, other grounds for retraction (e.g., plagiarism, data fabrication, ethical breaches, or concurrent submission to multiple venues, and the like) require a rigorous, judicious, and unhurried review. If misconduct, particularly ‘conscious negligence’ on the part of the author(s), is substantiated, then appropriate legal and professional sanctions must be duly enforced.

## Institutional Policy Recommendations

 Consequently, it is suggested that relevant oversight bodies (e.g., COPE, publishing councils, and ethics committees) develop and implement explicit guidelines that actively ‘*motivate authors to withdraw articles*’ when they become aware that their previously published findings are no longer credible. The formulation and enforcement of such policies will undoubtedly lead to *enhanced scientific clarity* while preventing the misdirection of the broader research community.

## Competing Interests

 The author declares no potential conflict of interests with respect to the research.

## Ethical Approval

 None.

## Funding

 The author received no financial support for the research.
